# Wastewater treatment bacteria show differential preference for colonizing natural biopolymers

**DOI:** 10.1007/s00253-024-13162-x

**Published:** 2024-05-06

**Authors:** Zongzong Liu, Onder Kimyon, Mike Manefield

**Affiliations:** 1 School of Chemical Engineering, University of New South Wales (UNSW), Sydney, 2052 Australia; 2 School of Civil and Environmental Engineering, University of New South Wales (UNSW), Sydney, 2052 Australia

**Keywords:** Biofilm, Chitin, Keratin, Cellulose, Lignocellulose, Lignin, Nitrogen, Activated sludge

## Abstract

**Abstract:**

Most reduced organic matter entering activated sludge systems is particulate (1–100-µm diameter) or colloidal (0.001–1-µm diameter), yet little is known about colonization of particulate organic matter by activated sludge bacteria. In this study, colonization of biopolymers (chitin, keratin, lignocellulose, lignin, and cellulose) by activated sludge bacteria was compared with colonization of glass beads in the presence and absence of regular nutrient amendment (acetate and ammonia). Scanning electron microscopy and quantitative PCR revealed chitin and cellulose were most readily colonized followed by lignin and lignocellulose, while keratin and glass beads were relatively resistant to colonization. Bacterial community profiles on particles compared to sludge confirmed that specific bacterial phylotypes preferentially colonize different biopolymers. Nitrifying bacteria proved adept at colonizing particles, achieving higher relative abundance on particles compared to bulk sludge. Denitrifying bacteria showed similar or lower relative abundance on particles compared to sludge.

**Key points:**

• *Some activated sludge bacteria colonize natural biopolymers more readily than others.*

• *Nitrifying bacteria are overrepresented in natural biopolymer biofilm communities.*

• *Biopolymers in wastewater likely influence activated sludge community composition.*

**Supplementary Information:**

The online version contains supplementary material available at 10.1007/s00253-024-13162-x.

## Introduction

Domestic wastewater treatment plants typically utilize a biological treatment process to convert particulate and dissolved forms of reduced organic matter into aggregated biomass that is removed by sedimentation and/or filtration (Metcalf and Eddy 2014). The biomass, referred to as activated sludge, is composed of biological flocculates, or aggregated cells embedded in a matrix of polymeric substances (saccharides, proteins, nucleic acids) (Maunders and Welch 2017).

Activated sludge flocculates harbor diverse microbial species including bacteria, eukaryotes, archaea, and viruses, with bacteria being by far the most abundant and responsible for most of the reduced organic matter oxidation occurring in wastewater (Metcalf and Eddy 2014). Bacterial communities in activated sludge are dominated by *Proteobacteria*, *Actinobacteria*, and *Bacteroidetes* with community composition underpinning wastewater treatment function (Wagner and Loy 2002; Wu et al. 2019; Palanisamy et al. 2022). Disfunction in floc formation, dictated by microbial community composition, results in failures in wastewater treatment plants (Metcalf and Eddy 2014).

Various processes are known to influence the microbial community composition of activated sludge including environmental conditions, biological interactions, and immigration from influent wastewater. Recent work by Dottorini et al. (2021) conclusively demonstrated that immigration from sewage has a dominant influence in shaping community composition in sludge. Here we explore the fundamental hypothesis that the abilities of microorganisms to colonize biopolymers in the form of particulate organic matter incumbent in wastewater may also play a role in shaping microbial community composition based on the following reasoning.

In wastewater treatment plants, primary sedimentation processes remove almost half of the influent biological oxygen demand. Particles of organic matter that are large (> 100 µm) and dense enough are separated from the wastewater by gravity. Particulate (1–100 µm) and colloidal (0.001–1 µm) organic matter that does not settle in primary sedimentation basins passes through to the secondary treatment units that convert particulate, colloidal, and dissolved organic matter into activated sludge flocs, carbon dioxide, and water. Up to 80% of the organic matter entering secondary treatment is particulate, so it stands to reason that colonization of particulate organic matter could influence microbial community composition (Metcalf and Eddy 2014). Despite the abundance of particulate organic matter entering secondary treatment, very little is known about its colonization by bacteria in wastewater treatment systems, including whether microorganisms show preferential colonization of different biopolymers.

This study describes the colonization of five biopolymers (chitin, keratin, cellulose, lignin, and lignocellulose) incubated in activated sludge over a 2-week period, including characterization of the bacterial communities formed. Cellulose is a glucose polymer and the most abundant organic polymer on Earth by mass (10^12^ tonnes produced year), mainly in the form of wood (Kaplan 1998; Dekker 1985). It is also the main component of toilet paper, accounting for approximately 30% of total suspended solids in domestic wastewater (Ahmed et al. 2019). Addition of chitin or chitosan derivatives to activated sludge improves settleability (Gooday 1994). Keratin is an amino acid polymer and the principal component of hair and wool. Hair shavings and wool fragments enter sewers from domestic bathroom and laundry facilities. Lignin is the second most abundant biopolymer on Earth, being a major structural component of wood and lignocellulose consisting of cellulose (50–60%), hemicellulose (25–35%), and lignin (Maki et al. 2009), likely entering wastewater as partially digested vegetable matter. Results reveal that specific microorganisms show clear preference with respect to colonization of different biopolymers suggesting this may influence community composition and therefore function in wastewater treatment plants.

## Materials and methods

### Chemicals and activated sludge samples

Chitin flakes (~ 100 µm) were purchased from Sigma-Aldrich (C9213-1 kg), human hair samples (length, 5-cm; diameter, ~ 100 µm) representing keratin were acquired from a local hair salon. Cellulose powder (~ 200 µm) from cottonseed and lignin alkali (370,959–100 G, 100 µm) were sourced from Sigma-Aldrich. Sawdust (1 mm) from hardwood was used as a lignocellulose source. Glass beads (2-mm diameter) were sourced from Enamel Warehouse Co. Before incubation in activated sludge, particulate organic matter samples were rinsed in 80% (v/v) ethanol, followed by extensive rinsing in MilliQ water for sterilization. Nylon mesh filter bags (50-µm mesh, SFBNM0110-5 pK) were purchased from Scintex. Sodium acetate, anhydrous (chem-supply, SA005-500 G) was supplied as an additional carbon and energy source, and ammonium acetate (Sigma-Aldrich, A1542-2.5 KG) was used as a supplementary nitrogen, energy, and carbon source. Activated sludge was sourced from a municipal wastewater treatment plant treating municipal sewage (Riverstone Wastewater Treatment Plant) located in Vineyard, Sydney, NSW. The activated sludge samples were collected from the aerobic unit process (aeration step) of a four-stage biological nutrient removal type treatment on Aug. 2020. The mixed liquor suspended solids (MLSS) of the activated sludge was 5250 mg/L measured by the standard method from American Public Health Association (Baird 2017).

### Activated sludge incubations

#### Keratin incubations in activated sludge

Keratin samples were incubated aerobically in three 1-L Schott bottles containing 500 mL activated sludge for 2 weeks, shaking at 200 rpm at 22 °C. After the addition of sterilized keratin in activated sludge, 1 mL activated sludge and 0.5-g keratin were sampled immediately and after 1 and 2 weeks from every bioreactor, for subsequent molecular analysis and surface observation by scanning electron microscopy (SEM). A 2-week incubation is a representative of sludge retention times (SRT) in domestic wastewater treatment plants. It was possible to directly retrieve hair samples from activated sludge.

#### Lignocellulose incubations in activated sludge with/without nutrients

Activated sludge used for lignocellulose incubations was divided into two groups. In the first group, 500 mL activated sludge was untreated while in the other group, activated sludge was amended with 2 mM ammonium acetate and 18 mM sodium acetate every 12 h as carbon, energy, and nitrogen source. Preliminary testing showed that this supplementation regime did not result in accumulation of either substrate. Particulate lignocellulose samples (0.2 g) were placed in nylon mesh bags with 50-µm pore size enabling passage of bacteria into the nylon bag while retaining the lignocellulose within. Nylon bags containing 0.2-g lignocellulose were incubated in activated sludge with and without nutrient amendment. At each time point (week 0, week 1, week 2), 1 mL activated sludge and 0.2-g lignocellulose samples were taken from reactors A, B, and C and stored at − 20 °C for further analysis. A pH of approximately 8 (+ / − 0.5 pH units) was maintained by adding 5–10 mL of 0.1 HCl every 2 to 3 days.

#### Chitin, cellulose, lignin, and glass incubations in activated sludge

Chitin powder (0.15 g), cellulose powder (0.2 g), lignin powder (0.2 g), and glass beads (0.5 g) were incubated in nylon bags submerged in activated sludge under nutrient amendment as above. Particulate organic matter (POM) and sludge samples were taken from triplicate reactors after 0-, 1-, and 2-week incubation and stored at − 20 °C for subsequent community analysis and SEM. pH in the activated sludge system was adjusted to 7.5–8.5 by adding 0.1 M HCl during a 14-day incubation.

### Microbial community characterization

To characterize microbial communities colonizing POM as well as monitor community succession in activated sludge, genomic DNA extraction was performed separately on individual POM samples (0.2-g wet weight) and activated sludge samples in triplicate reactors using QIAamp PowerFecal DNA Kit (Qiagen) according to the manufacturer’s protocol and stored at − 20 °C until further analysis.

The V4 region of 16S rRNA genes was amplified by PCR with a T100 ThermalCycler (Bio-Rad) using primers 515F (5′- GTG CCA GCM GCC GCG GTA A -3′)/806R (5′- GGA CTA CHV GGG TWT CTA AT -3′) (Caporaso et al. 2011). The 40 μL reaction is comprised of extracted DNA (2 μL of 2 μg/mL), 20 μL green 2 × Master Mix(Econotaq), 0.4 μL 515F primer (10 μM), 0.4 μL 806R primer (10 μM), and 17.2 μL DNA-free PCR-grade water, subject to the following cycling program: 3 min at 94 °C, then 35 cycles of 45 s at 94 °C, 1 min at 50 °C, 90 s at 72 °C, followed by 10 min at 72 °C, and storage at 4 °C until further analysis. PCR products were verified by gel electrophoresis.

PCR products were purified using the QIAquick PCR Purification Kit (Qiagen, 28,194) and sequenced on a multiplexed 2 × 250 bp and 2 × 300 bp using an Illumina MiSeq (Illumina, USA) at the Next Generation Sequencing Facility at Western Sydney University’s Hawkesbury Institute for the Environment (Sydney, Australia). The number of tags on each sample is higher than 30,000 to ensure the sequencing depth. The mean sequence quality (Phred Score) of sequencing read is 37. Sequence data of each sample were checked with FastQC (www.bioinformatics.babraham.ac.uk) and trimmed, analyzed on QIIME2 (https://qiime2.org) together using the dada2 pipeline against the Silva 132 reference database. After trimming, the number of filter reads on each sample exceeds 20,000.

### Scanning electron microscopy (SEM)

POM samples were removed from activated sludge after 0-, 1-, 2-week incubation and rinsed with filtered sludge supernatant to remove loosely attached cells. Samples were fixed with 2% (v/v) glutaraldehyde and 2.5% (v/v) paraformaldehyde for 2 h and then immersed in 0.1 M phosphate buffered saline at 4 °C overnight. After that, fixed samples were rinsed with 0.1 M phosphate buffered saline twice, followed by post-fixation in 0.1 M PBS containing 0.1% (w/v) osmium for 1 h. Samples were then subjected to sequential dehydration through ethanol dilutions between 30 and 100% (v/v), followed by submerging in reagents mixed by hexamethyldisilazane and ethanol with the ratios of 2:1 and 1:2 respectively for 20 min, and immersing in 100% hexamethyldisilazane until completely dry. Before imaging, platinum sputtering was performed with an Emitech K 550 sputter coater using Argon as an inert gas after placing treated organic matter samples on aluminum stubs (25-mm diameter). SEM imaging and analysis were performed on a NanoSEM 230 instrument at the Electron Microscopy Unit, UNSW. Electron beam voltage for imaging was typically 5 kV (Hazrin-Chong and Manefield 2012). A total of five pictures with same magnification (5000 ×) for each section were obtained randomly to calculate the number of cells distributed on different POM.

### Quantitative PCR (qPCR)

The abundance of 16S rRNA genes was quantified by qPCR on a CFX96 Real-Time PCR Detection System (BioRad). The 10 μL reactions contained 2 μL 100 × diluted or 1000 × diluted extracted DNA, 5 μL 2 × SsoFast EvaGreen Supermix (BioRad), 0.1 μL 1048F primer (5′-GTG STG CAY GGY TGT CGT CA-3′, 10 μM), 0.1 μL 1194R primer (5′-ACG TCR TCC MCA CCT TCC TC-3′, 10 μM), 0.1 μL bovine serum albumin (BSA) enzyme, and 2.7 μL DNA-free PCR-grade water (Horz et al. 2005).

Thermocycling involved an initial denaturation at 98 °C for 3 min, followed by 45 cycles of denaturation at 95 °C for 30 s, annealing at 58 °C for 50 s. Product specificity reflected by melting curve analysis was conducted with increments at 0.5 °C per 5 s from 55 to 95 °C. The internal standard calibration curve was made using *Escherichia coli* genomic DNA from 10 to 10^8^ copies. The *R*^2^ values of the standard curves was > 0.99, and the estimated amplification efficiency was between 95 and 104%. Standards, samples, and controls without DNA template were simultaneously run in triplicate in semi-skirted 96-well PCR plates (Scarlett et al. 2021).

### Quantification of acetate, ammonia, nitrite, and nitrate

Acetate (ethyl ester derivative) was quantified by gas chromatography (GC-FID) using a DB-FFAP column (30 m × 0.32 mm × 0.25 mm, Agilent Technologies). Sludge samples were centrifuged at 5000 rpm for 1 min to generate supernatant samples. Supernatant (500 μL), 200 μL 100% ethyl alcohol, and 200 μL undiluted sulfuric acid were mixed in GC vials for esterification, sealed with aluminum crimp caps, and incubated in a water bath (60 °C) for 45 min. Prior to injection into the gas chromatograph, 900 μL samples were incubated at 80 °C, 500 rpm, then 250 μL headspace samples were withdrawn by autosampler (Shimadzu AOC-5000 plus) and injected into a Shimadzu Plus GC-2010 with the flow rate at 500 μL/s. Samples were run at 40 °C for 6 min using helium as carrier gas.

Ammonia was quantified using the ammonia spectrophotometric assay kit (Megazyme) according to the manufacturer’s protocols and were read on a NanoDrop spectrophotometer at *λ* = 340 nm. Nitrite and nitrate quantification was conducted according to Miranda et al. (2001a). Vanadium trichloride (VCl_3_) stock was prepared by dissolving 400 mg VCl_3_ into 50 mL HCl (1 M) and stored at 4 °C in the dark for further use. Griess reagents consisted of 50 μL NEDD (0.1% w/v in ${{\mathrm{H}}}_{2}{\mathrm{O}}$) and 50 μL SULF (2% w/v in 5% HCl) premixed prior to addition to plates. For nitrate quantification, 100 μL samples or standards and 100 μL VCl_3_ were mixed in each well followed by addition of 100 μL Griess reagents and incubated for 45 min. Nitrite and nitrate standard solutions were serially diluted to 0.5, 1, and 2 mM. Absorbance was then measured at 540 nm using a micro-plater spectrophotometer (Shimadzu, UV-1800). Linear regression was conducted by the concentration of standard solution and absorbance at 540 nm to determine the amount of nitrate in samples. Nitrite measurements were the same as for nitrate except that VCl_3_ was replaced by water.

### Statistical analysis

Welch’s *T* test was used to test if the mean of 16S copy numbers associated with organic particles was significantly different before and after incubation in activated sludge (week 0 and week 2). The Shannon diversity index (*H*′) was calculated as follows:

$${H}^{\mathrm{^{\prime}}}=-\sum_{i=1}^{R}{P}_{i}{\mathrm{ln}}{P}_{i}$$

${P}_{i}$ refers to the proportion of species *i*, which is the number of species *i* divided by the sum of all species. *R* represents the total number of species present and $H\mathrm{^{\prime}}$ represents the calculated Shannon diversity index. Wilcoxon rank-sum test and Kruskal–Wallis test were employed in R software (v3.4.1). The taxonomic comparison at OTU level between data obtained from POM, glass beads, and activated sludge was performed using principal coordinates analysis (PCoA) and Permanova test from the Qiime package (v 1.80). Linear discriminant analysis effect size was examined using LEfSe 1.0 (Segata et al. 2011) with an LDA score filter value of 4.

## Results

### Activated sludge bacteria colonize particulate organic matter

**Fig. 1:**
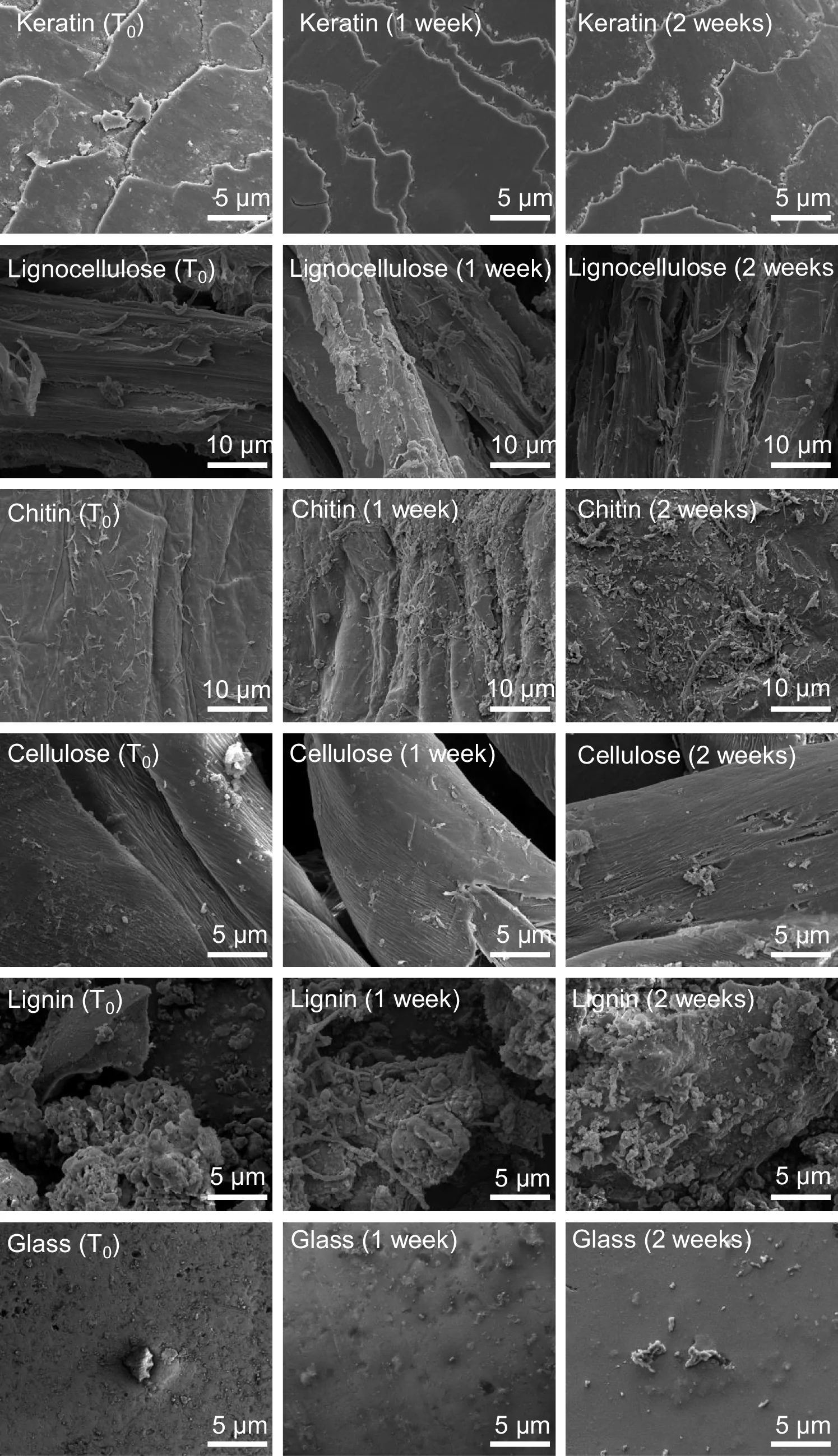
Scanning electron microscopy images of cells on particle surfaces. Particles include keratin, lignocellulose, chitin, cellulose, lignin, and glass after incubation in aerated activated sludge after 0, 1, and 2 weeks. Images of keratin are from incubations without nutrient amendment. Images from lignocellulose, chitin, cellulose, lignin, and glass are from incubations with nutrient amendment. Panels represent a surface area of approximately 1000–3000 µm^2^. Profuse colonization is apparent on chitin and lignin

**Fig. 2:**
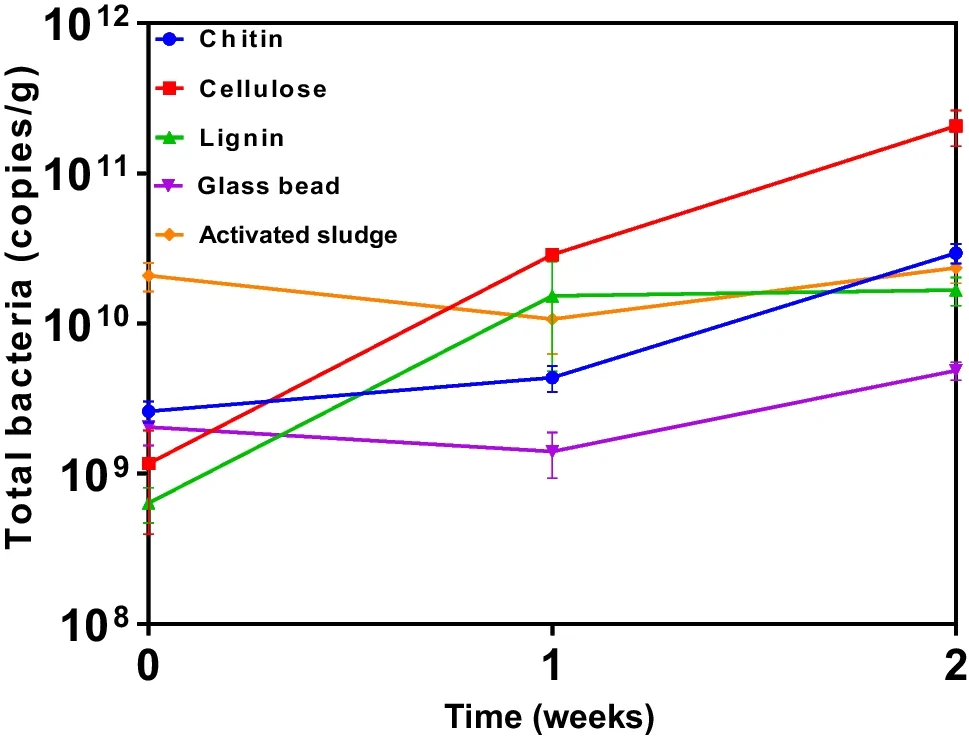
Abundance of bacteria (16S rRNA gene copies) on organic particles in activated sludge. Particles include chitin, cellulose, lignin, and glass beads in nutrient-amended activated sludge incubated with aeration for 2 weeks. The sludge data represents gene copies per milliliter. Significant increases in cell number attached to chitin, cellulose, lignin, and glass were observed from week 0 to week 2 (Welch two-sample *T* test; *P* = 0.003, 0.0003, 0.005, 0.001 respectively). There was no significant change in cell number in activated sludge over 2 weeks (*P* = 0.16). Data points represent the mean from triplicate incubations. Error bars represent standard deviation

To examine particle colonization in activated sludge, we initially incubated keratin and lignocellulose in shaking aerated activated sludge samples for 2 weeks (representative of sludge/solids retention time; SRT). In a second iteration, we incubated lignocellulose, chitin, cellulose, lignin, and glass beads in sludge with nutrient amendment. In both iterations, particles were recovered immediately after immersion (to assess attachment) and after 1 and 2 weeks of incubation (to assess colonization) and observed by scanning electron microscopy (SEM; Fig. 1). Based on microscopy, chitin followed by lignin and lignocellulose were preferred surfaces for colonization with the lowest cell counts per unit surface area observed on glass after 2 weeks (Table 1). Quantification of 16S rRNA gene copy number per gram of particulate matter by qPCR generally supported this (Fig. 2), although a discrepancy between SEM and qPCR was observed for cellulose colonization. There was scant evidence of colonization of cellulose from SEM while qPCR revealed cellulose to be carrying an order of magnitude higher biomass per gram compared to activated sludge, chitin, and lignin (Fig. 2).

**Table 1:** Cell density and Shannon diversity of bacterial communities in sludge and on particles

	Cell density ± StDv (cells/µm^2^)	Shannon diversity ± StDv
**Without nutrients**		
Activated sludge	-	5.23 ± 0.21
Keratin	5.3 (± 0.4) × 10^−2^	4.97 ± 0.41
Lignocellulose	5.0 (± 2.2) × 10^−2^	5.26 ± 0.18
**With nutrients**		
Activated sludge	-	4.77 ± 1.23
Lignocellulose	4.7 (± 1.4) × 10^−2^	4.11 ± 0.45
Chitin	1.4 (± 0.1) × 10^−1^	4.82 ± 1.09
Cellulose	3.0 (± 0.7) × 10^−2^	4.2 ± 1.37
Lignin	5.0 (± 0.6) × 10^−2^	4.58 ± 1.2
Glass	2.2 (± 0.6) × 10^−2^	5.13 ± 0.94

### Particle type influences bacterial community diversity and composition

DNA extracted from activated sludge and recovered biopolymers during the incubation was sequenced (16S rRNA gene amplicons) to assess changes in bacterial community composition. Table 1 reports Shannon diversity for each community after 2 weeks of incubation. Communities in incubations amended with nutrients (2 mM ammonium, 20 mM acetate) had lower diversity than those without nutrient amendment. Glass selected for the highest diversity in the presence of nutrients followed by chitin and lignin. The diversity of communities on cellulose and lignocellulose was more comparable with the sludge. The cellulose community had low diversity, suggesting colonization was dominated by specific bacterial lineages more so than the other particle types (Table 1).

**Fig. 3:**
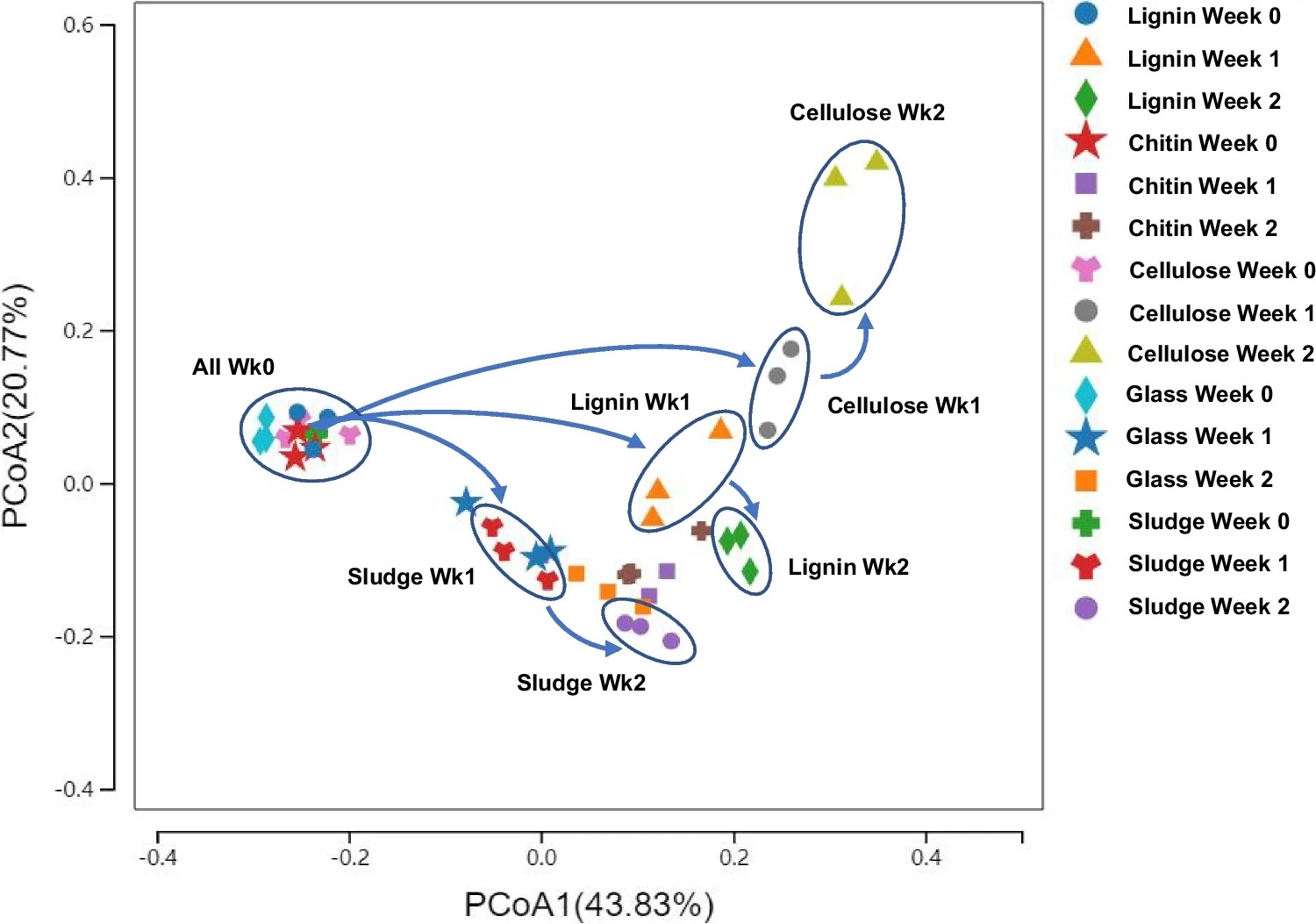
Principal coordinate analysis (PCoA) of microbial community sequencing data (16S rRNA gene). Communities are from activated sludge, lignin, chitin, cellulose, and glass surfaces immersed in nutrient amended sludge over 2 weeks. Communities in sludge and on surfaces were similar at time zero (immediately after immersion). All communities changed over time (*P* = 0.0001 based on Permanova test). Communities on chitin and glass beads resembled sludge communities. Communities on cellulose and lignin diverged from sludge and each other

Principal coordinate analysis (Fig. 3) revealed that the communities of bacteria that initially attached to particles immersed in activated sludge samples were similar to the sludge community. All communities shifted over the course of the incubations, including the activated sludge community (Fig. 3). Communities on glass and chitin surfaces resembled the sludge community throughout the incubation. Communities on lignin and cellulose diverged, indicating these surfaces selected for communities distinct from the bulk activated sludge. Keratin incubated in sludge in the absence of nutrient amendment also revealed divergence of the surface associated community from the sludge community (Fig. S1). These observations support the contention that specific types of organic particles can select for bacterial communities distinct from the sludge they are immersed in.

### Specific bacterial lineages show preference for colonization of organic particles

**Fig. 4:**
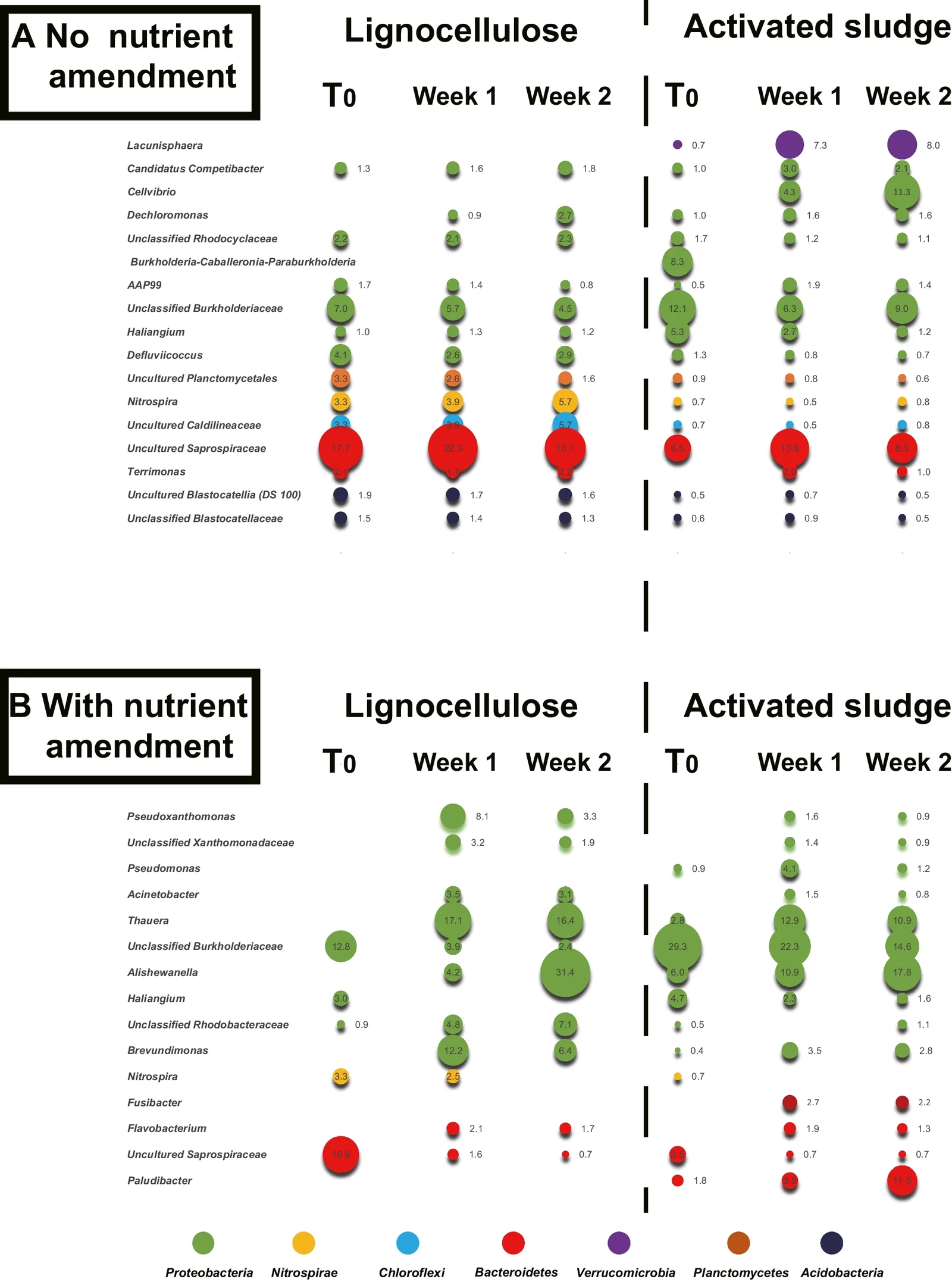
Relative abundance of bacterial lineages colonizing lignocellulose in activated sludge with or without nutrient amendment over 2 weeks. Sphere size represents the relative abundance (%) of bacterial lineages based on Illumina sequencing of 16S rRNA genes. Lineages with less than 1% relative abundance were not included. Phyla are color coded. Saprospiraceae, Burkholderiaceae, Caldilineaceae, and *Nitrospira* dominate lignocellulose colonization without nutrient amendment. Proteobacteria (*Thauera* and *Allishewanella*) dominate colonization with nutrient amendment

Linear discriminant analysis effect size (LEfSe) was used to identify bacterial species with the most significant differences (LDA score > 4) in relative abundance on biopolymers compared to bulk activated sludge. On lignocellulose, unclassified lineages within the *Saprospiraceae*, *Burkholderiaceae*, *Caldilineaceae* families and a *Nitrospira* lineage dominated colonization in the absence of nutrient amendment (Fig. 4). *Nitrospira* had a higher relative abundance on lignocellulose than in the sludge, implying selection for this nitrifying bacterium by lignocellulose particles. With nutrient amendment, proteobacterial lineages (*Thauera* and *Alishewanella*) dominated colonization of lignocellulose and relative abundance in the sludge, driving convergence with activated sludge community composition after a 2-week incubation (Figs. 3 and Fig. 4). A *Paulobacter* lineage increased in relative abundance in the sludge but did not appear on lignocellulose, again suggesting the particle surface selected for or against specific phylotypes (Fig. 4).

**Fig. 5:**
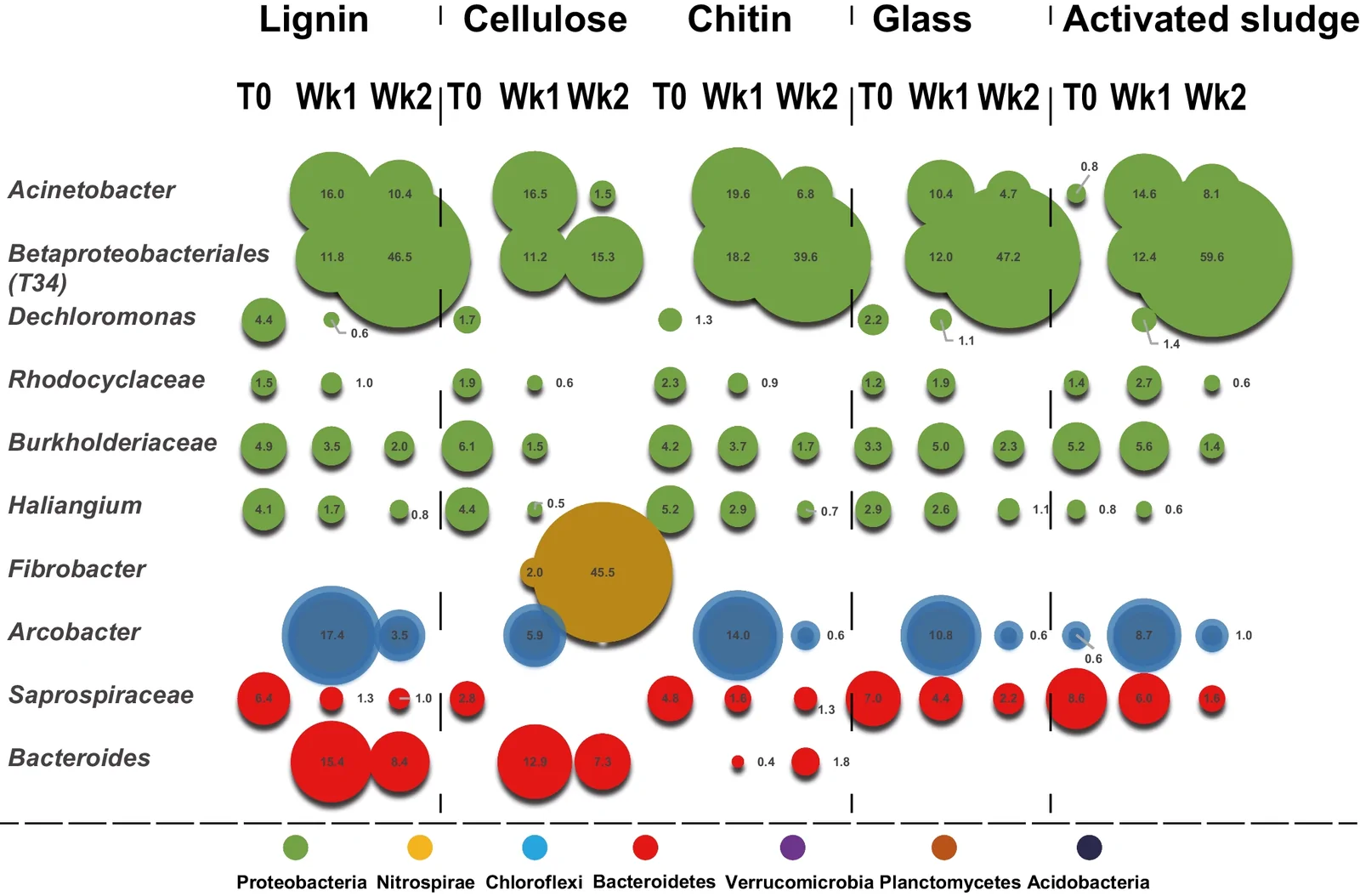
Relative abundance of bacterial lineages colonizing lignin, cellulose, chitin, and glass beads in activated sludge with nutrient amendment over 2 weeks. Sphere size represents the relative abundance (%) of bacterial lineages based on Illumina sequencing of 16S rRNA genes. Lineages with less than 1% relative abundance were not included. Phyla are color coded

An unclassified beta-proteobacterial and an *Acinetobacter* lineage dominated colonization of chitin, lignin, cellulose, and glass and dominated relative abundance in activated sludge in nutrient amended incubations (Fig. 5). These lineages appear to thrive under the incubation conditions in the bulk sludge and attached to the amended particles. The functional role of the beta-proteobacterial lineage (T34) with no cultivated close relatives is worthy of further investigation given its high relative abundance. Conversely, a *Bacteroides* lineage became prominent specifically on lignin and cellulose surfaces (less so on chitin), while it was not observed on glass or in the sludge, consistent with a preference for organic particle colonization (Fig. 5). A *Fibrobacter* lineage within the *Planctomycetes* phylum dominated colonization of cellulose, while remaining below detection on all other surfaces and in activated sludge, indicating strong specificity for cellulose colonization (Fig. 5). This lineage was likely responsible for the high cell abundance measured by qPCR (Fig. 2) and the low diversity metric (Table 1). The community on glass was very similar to the community in the sludge in which the glass beads were immersed. These observations support the assertion that specific bacterial lineages in activated sludge have specific competencies in colonization of particulate organic matter.

The community divergence observed with keratin in the absence of nutrient amendment was driven by the increasing dominance of an *Aquabacterium* lineage from < 1 up to 15.7% relative abundance on the keratin surface, while it remained below 1% relative abundance in sludge, implying selection by the surface (Supplementary information).

### Colonization of lignocellulose by nitrifying and denitrifying bacteria

**Fig. 6:**
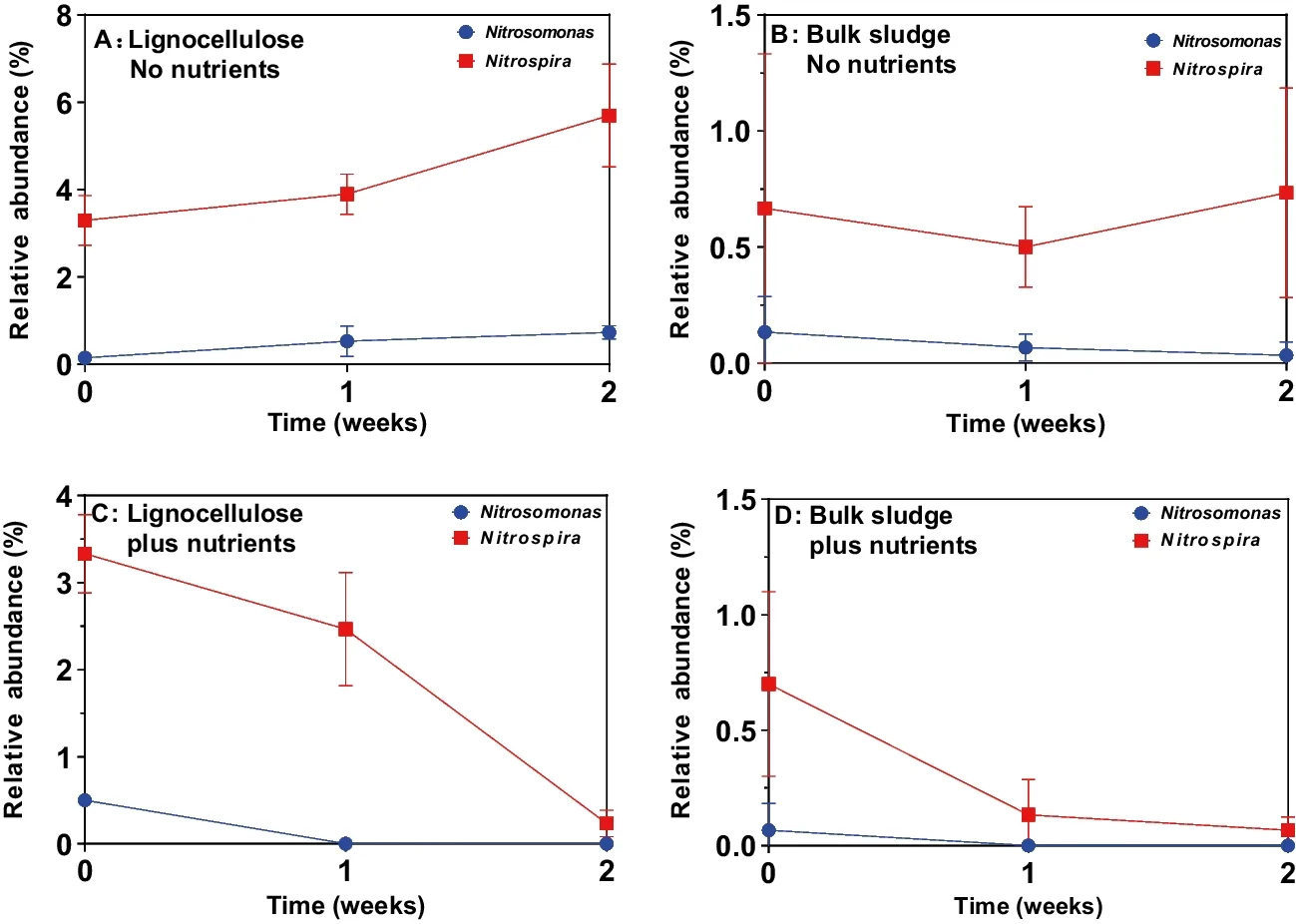
Nitrifying bacteria colonizing lignocellulose in activated sludge in the presence and absence of nutrients. Relative abundance of *Nitrospira* (nitrite oxidation) and *Nitrosomonas* (ammonia oxidation) on lignocellulose (**A**, **C**) and in bulk sludge (**B**, **D**) and in the absence (**A**, **B**) or presence (**C**, **D**) of nutrient amendment. Error bars represent standard deviation from three independent incubations

The relative abundance of nitrifying bacterial lineages *Nitrospira* and *Nitrosomonas* in lignocellulose-associated communities immersed in activated sludge was compared with the sludge (Fig. 6). *Nitrospira* (nitrite oxidation) had higher relative abundance than *Nitrosomonas* (ammonia oxidation) in sludge and on lignocellulose. After 2 weeks of incubation, both nitrifying lineages were more abundant (five to tenfold) on lignocellulose than in sludge with or without nutrient amendment indicative of competency in colonizing this surface. This observation suggests particulate organic matter in sludge can select for functionally important lineages. Nutrient amendment resulted in a decrease in relative abundance of both lineages over the incubation in sludge and on particulate organic matter owing to mass proliferation of other lineages.

**Fig. 7:**
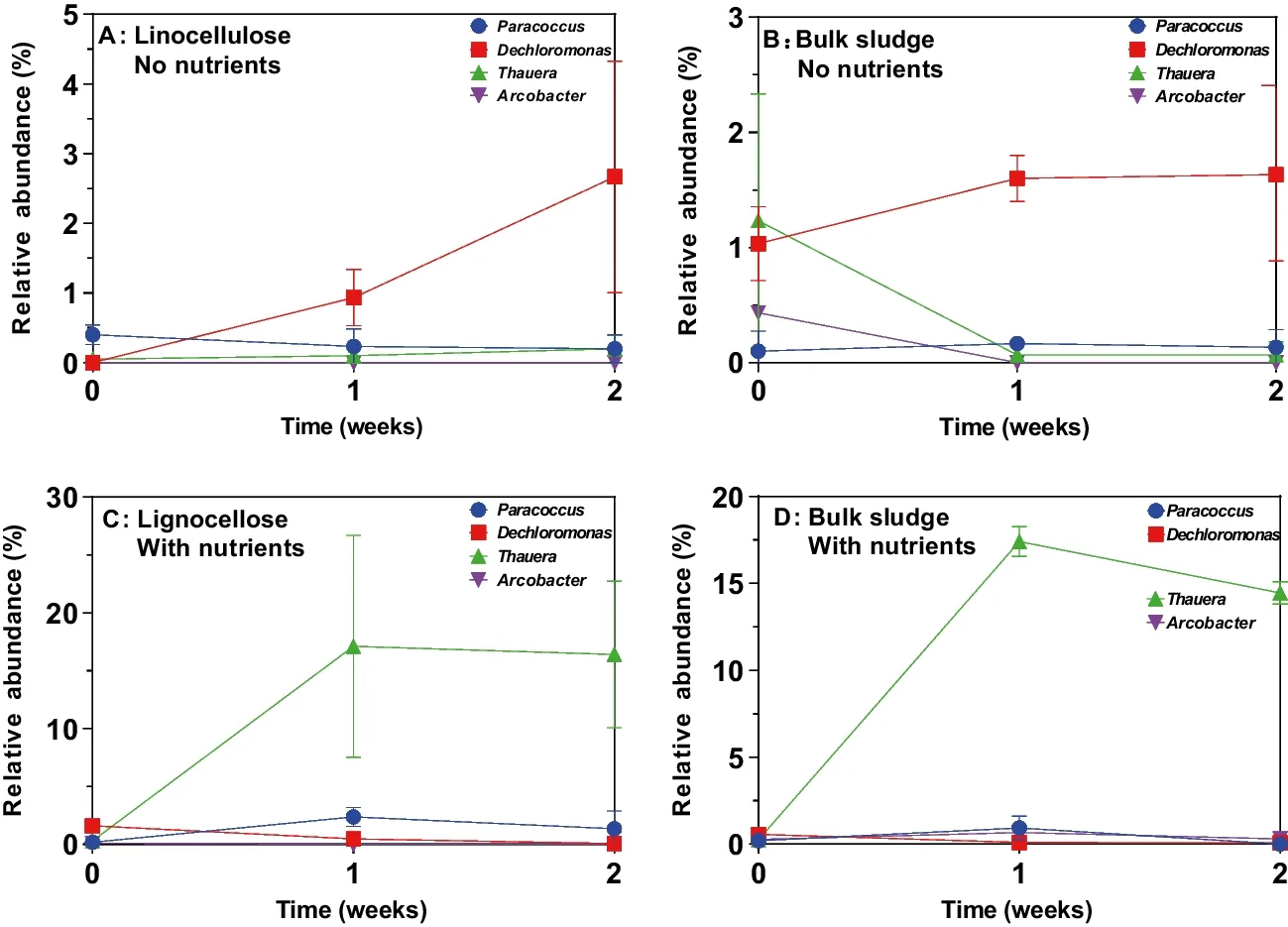
Relative abundance of known denitrifying bacteria (*Paracoccus*, *Dechloromonas*, *Thauera*, and *Arcobacter*) on lignocellulose and in activated sludge in the presence and absence of nutrients. Relative abundance on lignocellulose (**A**, **C**) and in bulk sludge (**B**, **D**) and in the absence (**A**, **B**) or presence (**C**, **D**) of nutrient amendment. Error bars represent standard deviation from three independent incubations

Seventeen known denitrifying bacterial lineages (McIlroy et al. 2016) were observed in the activated sludge sequencing data, eight of them being *Betaproteobacteria* (Table 2). Six of the 17 denitrifying lineages observed in the sludge were not observed above 0.1% relative abundance on any of the particulate organic matter tested, including all three nitrate reducing representatives of the Firmicutes phylum observed. *Dechloromonas* was the only known nitrate reducing lineage observed above 0.1% relative abundance on all particles after incubation. *Thauera*, *Zoogloea*, *Pseudomonas*, *Paracoccus*, and *Arcobacter* were also commonly observed denitrifying bacteria associated with particulate matter. *Dechloromonas* generally dominated in the absence of nutrients (up to 2.7%) and *Thauera* dominated with nutrient amendment (up to 17%) (Fig. 7).

**Table 2:** Denitrifying bacteria identified in biofilms on POM in activated sludge

	**Without nutrients**		**With nutrient amendment**				
**Genus**	Keratin	Lignocellulose	Lignin	Cellulose	Chitin	Glass	Lignocellulose
*Rhizobium*	< 0.1%	< 0.1%	0.1–1%	0.1–1%	0.1–1%	0.1–1%	0.1–1%
*Methylobacterium*	< 0.1%	< 0.1%	< 0.1%	< 0.1%	< 0.1%	< 0.1%	< 0.1%
*Paracoccus*	0.1–1%	0.1–%	0.1–1%	< 0.1%	0.1–1%	0.1–1%	> *1*%
*Acidovorax*	0.1–1%	< 0.1%	0.1–1%	< 0.1%	0.1–1%	0.1–1%	0.1–1%
*Comamonas*	< 0.1%	< 0.1%	0.1–1%	< 0.1%	0.1–1%	0.1–1%	0.1–1%
*Aquaspirillum*	< 0.1%	< 0.1%	< 0.1%	< 0.1%	< 0.1%	< 0.1%	< 0.1%
*Azospira*	0.1–1%	< 0.1%	< 0.1%	< 0.1%	< 0.1%	< 0.1%	< 0.1%
*Azovibrio*	< 0.1%	< 0.1%	< 0.1%	< 0.1%	< 0.1%	< 0.1%	< 0.1%
*Dechloromonas*	0.1–1%	> *1*%	> *1*%	0.1–1%	0.1–1%	> *1*%	0.1–1%
*Thauera*	0.1–1%	0.1–1%	0.1–1%	< 0.1%	0.1–1%	0.1–1%	> *1*%
*Zoogloea*	> *1*%	< 0.1%	0.1–1%	0.1–1%	0.1–1%	0.1–1%	< 0.1%
*Pseudomonas*	0.1–1%	< 0.1%	0.1–1%	0.1–1%	0.1–1%	0.1–1%	0.1–1%
*Arcobacter*	0.1–1%	< 0.1%	0.1–1%	> *1*%	0.1–1%	> *1*%	< 0.1%
*Bacillus*	< 0.1%	< 0.1%	< 0.1%	< 0.1%	< 0.1%	< 0.1%	< 0.1%
*Trichococcus*	< 0.1%	< 0.1%	< 0.1%	< 0.1%	< 0.1%	< 0.1%	< 0.1%
*Staphylococcus*	< 0.1%	< 0.1%	< 0.1%	< 0.1%	< 0.1%	< 0.1%	< 0.1%
*Chryseobacterium*	0.1–1%	< 0.1%	< 0.1%	< 0.1%	< 0.1%	< 0.1%	0.1–1%

Italics denote highest relative abundance (>1%)

## Discussion

Human civilization is heavily dependent on activated sludge systems in wastewater treatment plants to process primary treated domestic and industrial wastewater. The composition of activated sludge with respect to microbial lineages has been extensively studied, and there is a general understanding of the functional roles the most abundant lineages play given their genetically encoded catalytic abilities. Less progress has been made in predicting or rationally manipulating microbial community composition and function in activated sludge systems based on fundamental knowledge of the resident microbes. There remains a lack of understanding of activated sludge flocculate formation processes and the relationship between these processes and specific microbial lineages.

Typical domestic wastewater contains 100–300 mg/L total suspended solids (TSS; the fraction removed by a 1.2-µm filter) and 100–300 mg/L biological oxygen demand (BOD). Primary sedimentation typically removes 50–70% TSS and 40–50% BOD. The remaining 60–180 mg/L TSS and 45–135 mg/L BOD enters activated sludge systems for secondary treatment (Metcalf et al., 2014). This TSS figure does not include colloidal particles in the 0.001–1-µm size range (10^8^–10^12^ particles/mL) and is thus an underestimate. Given that immigration is the major driver of community composition in activated sludge (Dottorini et al. 2021) and that the majority of the reduced organic matter entering an activated sludge unit is particulate rather than dissolved, the ability of bacteria to colonize particulate organic matter entering wastewater treatment plants becomes a likely determinant of community composition in wastewater treatment plants. Depsite this, there has never been an analysis of bacteria in wastewater colonizing particulate organic matter.

**Fig. 8:**
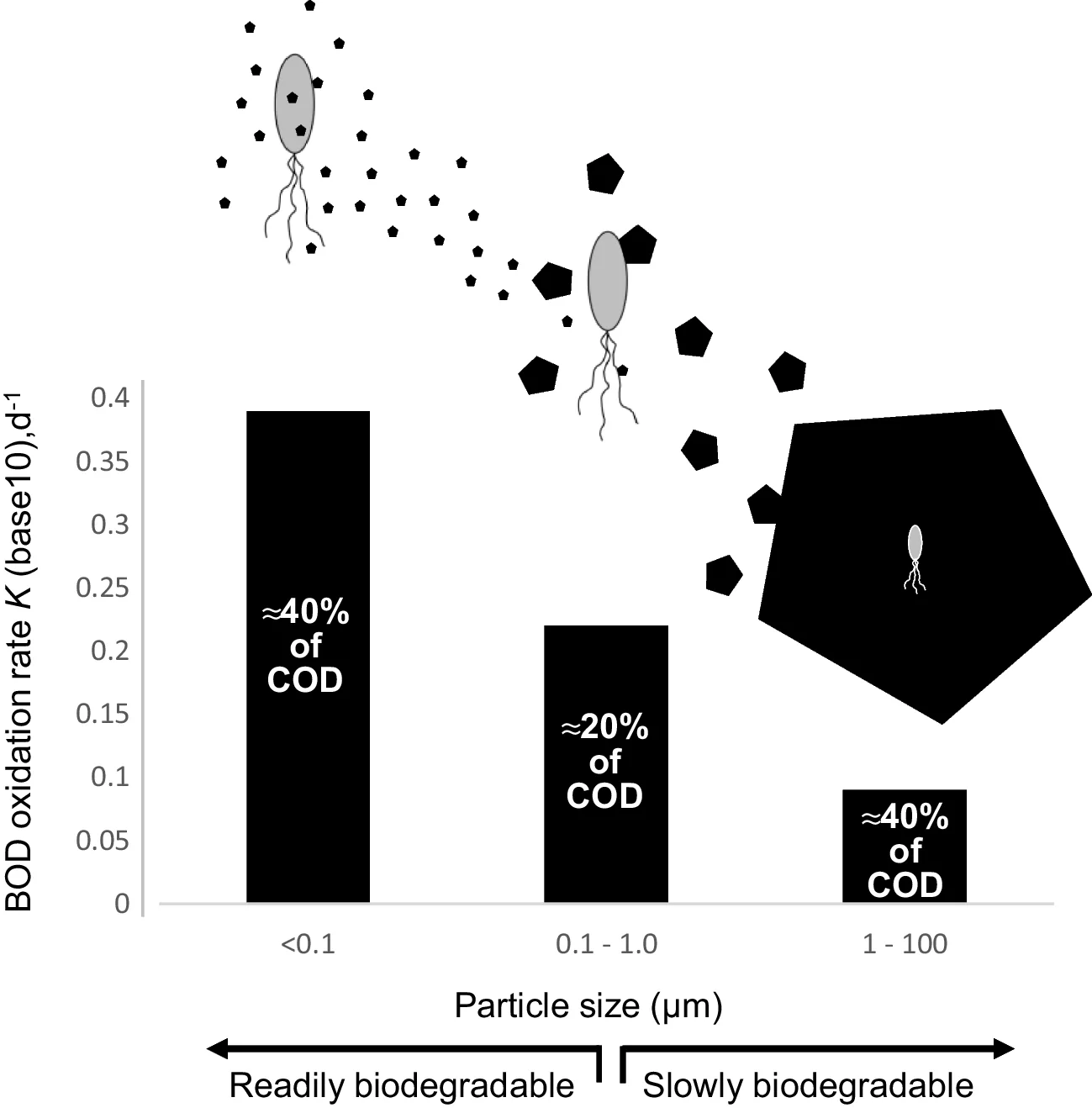
Relationship between particle size and biodegradability (BOD oxidation rate) of particles of organic matter in secondary treatment influent. Graphic compares particle size to bacterial cell size (width 1 µm). Approximate contribution of particle size fractions to chemical oxygen demand is shown on bars (Levine et al. 1985)

Figure 8 shows the biodegradability of particles in three size categories (< 0.1 µm, 0.1–1 µm, 1–100 µm) in primary treated wastewater and relates the particle sizes to the size of bacteria. Particles larger than 100 µm in diameter, representing approximately half the BOD in domestic wastewater, are typically removed through primary sedimentation and do not enter activated sludge systems. The figure also shows the proportion of chemical oxygen demand represented by particles in the three categories. When activated sludge settles in secondary clarifiers, the effluent wastewater no longer contains the dissolved or particulate BOD, which has either been biologically oxidized or entrained in the sludge matrix which settles out. This study is predicated on the hypothesis that slowly biodegradable particles (the 1–100-µm diameter range, Fig. 8) are colonized by bacteria in activated sludge, making up a large proportion of return activated sludge that adds continuity to the community composition of sludge (Levine et al. 1985).

The SEM, qPCR, and sequencing data presented show that particulate organic matter immersed in activated sludge is, unsuprisingly, colonized by bacteria. Evidence suggests that the nature of the particle influences the rate of colonization and the specific lineages of bacteria most successful in colonization. For example, the rate of colonization of lignin and cellulose was faster than for glass beads and chitin, and the bacterial communities assembling on lignin and cellulose diverged from activated sludge while those on glass and chitin did not.

There were several obvious examples of specific bacterial lineages with higher relative abundance on particular particles than in the sludge. These include *Fibrobacter* on cellulose, *Bacteroides* on lignin, cellulose and chitin, and *Nitropsira* and *Alishewanella* on lignocellulose. Clearly, particulate organic matter selects for specific bacterial lineages in activated sludge. This suggests that the type and concentrations of particulate organic matter, representing approximately half the chemical oxygen demand entering secondary treatment systems, can influence the bacterial community composition of activated sludge. The qPCR data shown in Fig. 2 indicated that after 2 weeks, chitin and lignin carried a similar 16S rRNA gene quantity per gram as activated sludge does per mL. For cellulose, this was an order of magnitude higher. Taken together, these data suggest the nature of the particulate organic matter in activated sludge could play a role in dictating sludge function (e.g., reducing oxygen demand and sludge settling properties).

Extensive research efforts have been directed towards colonization of membranes (polyethersulfone, polytetrafluoroethylene, polyvinylidene fluoride) and plastic carriers (polyethylene, polyurethane, polypropylene), used in membrane and moving bed reactors for wastewater treatment (Lee et al. 2014; Felföldi et al. 2015). Biofilms associated with microplastics (Kelly et al. 2021) and sponge loofah (Dang et al. 2020) have also been characterized. Use of different sludge samples, microbial community characterization techniques, experimental designs, and incubation conditions make direct comparisons difficult, but conclusions on diversity and divergence of biofilm communities from sludge composition observed here are generally supported.

*Fibrobacter* lineages have previously been observed in activated sludge and are well known for cellulose attachment and degradation (Ransom-Jones et al. 2012), but data presented here suggests they dominate cellulose colonization, and possibly degradation, in activated sludge. Cellulose inputs to sewage include incompletely digested vegetable matter and toilet paper. SEM analysis underestimated the quantity of cells attached to cellulose. Possible reasons include the biomass being internal to the cellulose structure and therefore unseen, the SEM sample preparation protocol being unsuitable for this genus, loose association based on its adhesion mechanism, or by chance the randomly selected SEM images missed colonization hotspots. *Fibrobacter succinogenes* adheres to cellulose by increasing the protein content at the cell surface on exposure to cellulose (vs growth on glucose) making the cell more hydrophobic. Of the 185 cell envelope associated proteins identified in *Fibrobacter succinogenes*, 25 are thought to be involved in cellulose adhesion (e.g., fibro-slime domain protein), degradation (e.g., cellulases, endoglucanase, glycoside hydrolase), and degradation product uptake (e.g., substrate transporters) (Raut et al. 2015).

A *Bacteroides* lineage proved adept at colonizing lignin and cellulose, while remaining at low relative abundance in activated sludge. *Bacteroides* lineages are abundant anaerobic inhabitants of cow rumen and the human gastrointestinal tract. They are known to encode diverse enzyme activities and play important roles in degradation of diverse plant polysaccharides, attracting extensive interest in gut health, rumen ecology, and biofuels applications (Chukwuma et al. 2021; Flint et al. 2012). A functional role for *Bacteroides* in particulate organic matter degradation in wastewater treatment has not previously been mentioned in the literature, and little is known about how *Bacteroides* colonizes organic particles. Extensive literature exists on a role for *Bacteroides* in antibiotic resistance determinant proliferation in wastewater treatment systems (Niestępski et al. 2020). This may be influenced by the lignin and cellulose content of the wastewater.

*Nitrospira* was observed at higher relative abundance on lignocellulose than in activated sludge in the absence of nutrient amendment. *Nitrospira* lineages play key roles in nitrification in wastewater treatment, therefore potential exists to increase the relative abundance of *Nitrospira* through amendment with lignocellulose or exploitation of lignocellulose-colonizing abilities. They have not previously been associated with lignocellulose degradation but an increase in abundance of *Nitrospira* in constructed wetlands containing lignocellulosic material has been reported (Wang et al. 2016). In contrast, denitrifying bacteria showed limited disposition to preferentially colonize particulate oragnic matter. Known denitrifying *Firmicutes* in particular were underrepressented on surfaces, possibly related to the relatively short incubation administered here. *Firmicutes* are often well represented in environmental biofilms.

These findings suggest there is potential to manipulate the microbial composition and hence functional properties of activated sludge through particle amendment or modification of the surface properties of inorganic carriers. For example, lignocellulose was shown to support increased relative abundance of bacteria involved in nitrification, namely *Nitrospira*. The provision of surfaces for colonization in wastewater treatment has, of course, been a common practice for as long as wastewater treatment has existed. Trickling filters are a foundational technology, and fixed or moving bed bioreactors are in widespread use today. Mineral or plastic carriers are used to retain biomass (Naz et al. 2013; Khatoon et al. 2014), support robust biomass activity and resilience, and to facilitate diffusion and exchange of oxygen, nutrients, and metabolic products. Their potential utility as a tool to rationally manipulate or design microbial communities to meet performance requirements of a given industrial or domestic wastewater stream may have not yet been fully realized.

## Supplementary Information

Below is the link to the electronic supplementary material.

Supplementary file1 (PDF 225 KB)

## Data Availability

The datasets generated during and/or analyzed during the current study are available in the NCBI Sequence Read Archive (SRA) under the BioProject ID PRJNA813021.
