# Supplementary material for: Wastewater treatment bacteria show differential preference for colonizing natural biopolymers
Source: Appl Microbiol Biotechnol. 2024 May 6;108:321. doi: 10.1007/s00253-024-13162-x (PMC11599380; doi:10.1007/s00253-024-13162-x)
Supplement: Supplementary file 1 [file 253_2024_13162_MOESM1_ESM.pdf]

Supplementary information

Applied Microbiology and Biotechnology

Title: Wastewater treatment bacteria show differential preference for colonizing natural biopolymers

Zongzong Liu[a], Onder Kimyon[b], Mike Manefield[b] *

[a]School of Chemical Engineering, University of New South Wales (UNSW), Sydney, 2052, Australia.

[b]School of Civil and Environmental Engineering, University of New South Wales (UNSW), Sydney, 2052, Australia.

*Corresponding author: Mike Manefield

School of Civil and Environmental Engineering,

University of New South Wales (UNSW)

Sydney, 2052, Australia.

Email: manefield@unsw.edu.au

Phone +61405477066

Supplementary Figure S1

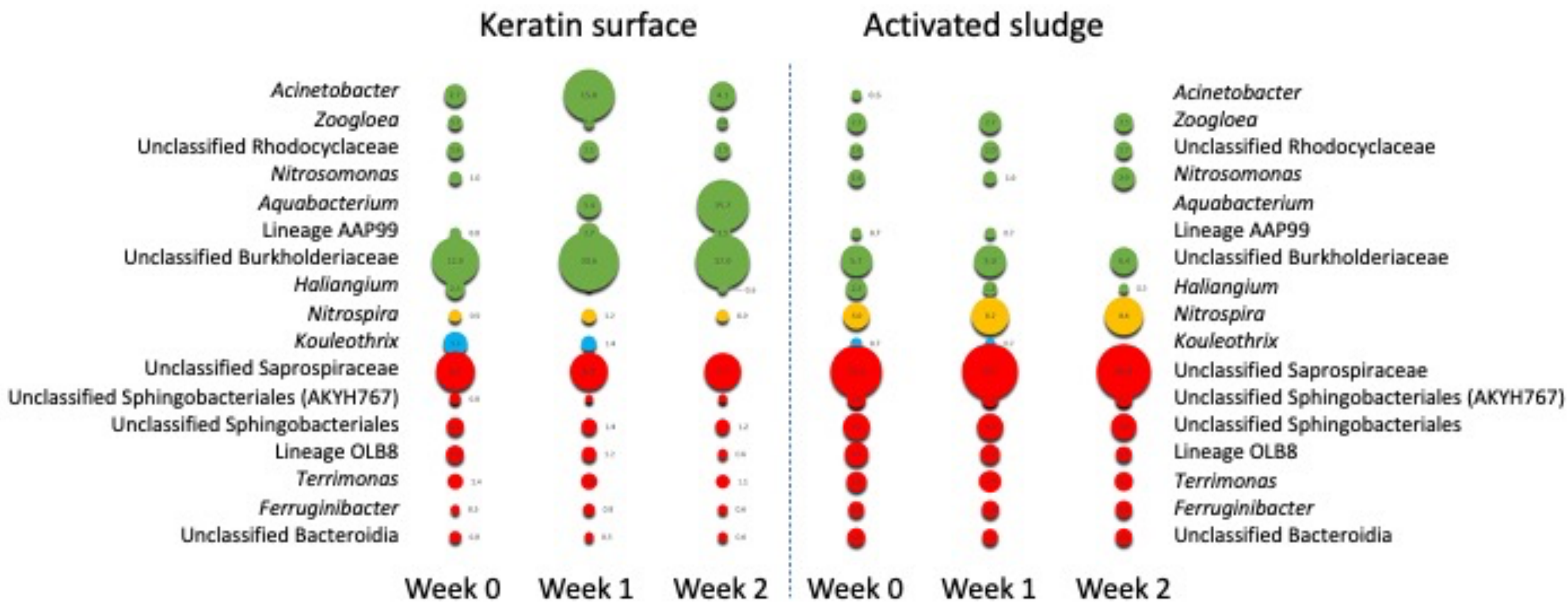

Figure S1. Bacterial lineages colonising keratin in activated sludge without nutrient amendment over two weeks based on Illumina sequencing of 16S rRNA gene (showing lineages with >0.1% relative abundance). Bubble sizes and figures represent the relative abundance (%) of bacterial lineages and different colors resemble the phylum to which bacterial lineage belongs. An *Aquabacterium* and an unclassified *Burkholderiaceae* lineage dominated colonization of keratin.
